# In the quest for new targets for pathogen eradication: the adenylosuccinate synthetase from the bacterium *Helicobacter pylori*

**DOI:** 10.1080/14756366.2018.1506773

**Published:** 2018-09-07

**Authors:** Ante Bubić, Natalia Mrnjavac, Igor Stuparević, Marta Łyczek, Beata Wielgus-Kutrowska, Agnieszka Bzowska, Marija Luić, Ivana Leščić Ašler

**Affiliations:** a Division of Physical Chemistry, Ruđer Bošković Institute, Zagreb, Croatia;; b Department of Chemistry and Biochemistry, Faculty of Food Technology and Biotechnology, University of Zagreb, Zagreb, Croatia;; c Division of Biophysics, Institute of Experimental Physics, Faculty of Physics, University of Warsaw, Warsaw, Poland;; d Department of Bacterial Genetics, Faculty of Biology, Institute of Microbiology, University of Warsaw, Warsaw, Poland

**Keywords:** *Helicobacter pylori*, adenylosuccinate synthetase, enzyme kinetics, enzyme inhibition, oligomeric state, analytical ultracentrifugation

## Abstract

Adenylosuccinate synthetase (AdSS) is an enzyme at regulatory point of purine metabolism. In pathogenic organisms which utilise only the purine salvage pathway, AdSS asserts itself as a promising drug target. One of these organisms is Helicobacter pylori, a wide-spread human pathogen involved in the development of many diseases. The rate of H. pylori antibiotic resistance is on the increase, making the quest for new drugs against this pathogen more important than ever. In this context, we describe here the properties of H. pylori AdSS. This enzyme exists in a dimeric active form independently of the presence of its ligands. Its narrow stability range and pH-neutral optimal working conditions reflect the bacterium’s high level of adaptation to its living environment. Efficient inhibition of H. pylori AdSS with hadacidin and adenylosuccinate gives hope of finding novel drugs that aim at eradicating this dangerous pathogen.

## Introduction

In order to effectively inhibit an enzyme, one should know its properties and mechanism of action in the greatest possible detail. If the enzyme in question (adenylosuccinate synthetase (AdSS)) is a potential drug target against a wide-spread human pathogen (*Helicobacter pylori*), the elaborate investigation of this enzyme is all the more important.

AdSS (EC 6.3.4.4) catalyses the first step in the biosynthesis of AMP from IMP, generating adenylosuccinate:

$$\text{IMP}+\text{Asp}+\text{GTP}({\text{Mg}}^{2}{}^{+})↔\text{adenylosuccinate}+\text{GDP}+P_{i}$$

In the second step, adenylosuccinate lyase cleaves adenylosuccinate to form AMP. AdSS operates at a branch point of the *de novo* synthesis of purines and the purine nucleotide cycle, the so-called salvage pathway, which makes this enzyme a challenging subject to study^1^. AdSS activity has been observed in all the tissues investigated, except in erythrocytes^1^. There are over 700 reviewed protein entries for the gene name “*purA*” in the UniProt database (July 2018) from all domains of life. Two AdSS enzymes were identified in vertebrates: acidic (pI ∼ 6) and basic (pI ∼ 9), presumably the former associated with the biosynthesis of purines and the latter with the purine nucleotide cycle^2^. Regulation of activity of these two isoforms is complex and different (as judged by different reactions with inhibitors), and is dependent on the isozyme content and levels in a given tissue, as well as substrate and product levels^1^. As AdSS operates at a regulatory point in the metabolism of purines, its substrate binding sites are quite specific^3^.

Bacterial AdSS enzymes resemble eukaryotic acidic isozymes in their properties^3^. AdSS from *Escherichia coli* is probably the most investigated of the kind. This protein exists in solution as a mixture of monomers (47 kDa) and dimers (2 × 47 kDa), with *K*_d_ = 10 µM for dimer dissociation^4^. In the presence of ligands practically all of the protein is in the form of dimers^4^. As monomer concentrations of AdSS are estimated to 1 µM *in vivo*, the enzyme may be activated by substrate-induced dimerisation^5^. Initial-rate kinetic studies by Rudolph and Fromm^6^ showed that the *E. coli* AdSS kinetic mechanism is rapid-equilibrium random. This type of mechanism was proven for several other AdSS enzymes^1^. The only characterised AdSS differing so far is the one from *Plasmodium falciparum*, reported to have ordered substrate binding^7^.

Numerous compounds have been investigated as potential inhibitors of *E. coli* AdSS, some of them listed in Supplementary Table S1. Mostly product/feedback inhibition was studied (AMP^8^, adenylosuccinate^6^^,^^8^, GDP^6^^,^^8^, GMP^8^), as well as stringent response (guanosine 3′,5′-bis(diphosphate) – ppGpp^9^, guanosine 5′-diphosphate 2′:3′-cyclic monophosphate – ppG2′:3′p^10^). Among other tested compounds, some proved to be potent inhibitors, such as the herbicides hydantocidin monophosphate^3^^,^^11^ and hadacidin^11^^,^^12^, and especially the hybrid inhibitor obtained by linking these two molecules *via* a C_3_ methylene chain, which gave an IC_50_ of 0.043 µM for *E. coli* AdSS^11^. Twenty-four 3D-structures of *E. coli* AdSS are deposited in Protein Data Bank (PDB) (July 2018), and most of them comprise complexes of the enzyme with the inhibitors mentioned above (usually in combination with substrates). However, only a few structures of AdSS from other microorganisms are available (unpublished, structures deposited in PDB) and the possible differences in active sites interactions are yet unknown.

The bacterium *H. pylori* represents a serious threat to human health. Even before its identification by Marshall and Warren^13^ it was suspected as a causative agent of gastritis, ulcer, and gastric cancer. Today it is proven to be involved in the development of several diseases, such as chronic active gastritis, peptic ulceration, gastric adenocarcinoma, and gastric mucosa-associated lymphoid tissue lymphoma^14^. In 1994 it was classified as a class I human carcinogen^15^. It is estimated that about half of the world’s population is infected with this pathogen and its prevalence goes up to ∼80% in several countries, like Nigeria, Serbia, Nicaragua, and Ecuador^16^, but also Latvia, Republic of Korea, and Japan (however, mainly in older subjects)^17^. Standard triple therapy for *H. pylori* infection consists of a proton pump inhibitor, amoxicillin, and clarithromycin^18^. However, with growing resistance to antimicrobial agents in *H. pylori* (up to 60% for clarithromycin in Korea^19^), new antibiotics are included, like metronidazole, levofloxacin, sitafloxacin, and rifabutin^18^. With a general increase in the consumption of antibiotics, the number of resistant strains is also expected to rise, so the search for novel targets for antimicrobial drugs is of utmost importance.

When the genome of *H. pylori* was published^20^, it opened possibilities for studying the physiology of this pathogen in search of promising drug targets^21^. It was observed that many of the redundant metabolic pathways are missing, among them the *de novo* synthesis of purines^22^. The fact that *H. pylori* relies exclusively on the salvage pathway for the acquisition of purines, encouraged studies of proteins involved in the purine salvage pathway (similarly as in e.g. parasitic protozoa^23^). Liechti and Goldberg^24^ investigated the growth of *H. pylori* mutants with deletions of purine salvage pathway enzymes on media supplemented with various purine nucleosides and bases and confirmed the essentiality of this metabolic pathway. They found that the Δ*purA* mutant (missing the gene *purA* encoding for AdSS) is capable of growth only in a medium supplemented with adenine or adenosine and that its growth was significantly retarded in nutrient-rich medium, thus indicating the essentiality of AdSS for the salvage pathway^24^. However, no records are available on the characterisation of this enzyme, yet.

In our work, we have turned our attention to less investigated enzymes from the purine salvage pathway of *H. pylori*, in hope of identifying possible new drug targets for the eradication of this persistent pathogen. Previously we described the catalytic properties and 3D-structure of purine nucleoside phosphorylase (PNP, EC 2.4.2.1)^25^^,^^26^ from this organism, and in this article we present the biochemical and kinetic characterisation of *H. pylori* AdSS (UniProt ID: P56137), along with its inhibition by a potent AdSS inhibitor, hadacidin, and the reaction product, adenylosuccinate.

## Materials and methods

### Materials

Substrates for AdSS: L-aspartic acid monosodium salt monohydrate (Asp), inosine 5′-monophosphate disodium salt hydrate (IMP) and guanosine 5′-triphosphate sodium salt hydrate (GTP) were purchased from Sigma (St. Louis, MO). Inhibitors of AdSS: hadacidin was purchased from Anji Biosciences (Hyderabad, India) and adenylosuccinate from Santa Cruz Biotechnology Inc. (Dallas, TX). Restriction enzymes and T4 DNA ligase used in DNA manipulations were purchased from New England BioLabs (Ipswich, MA). Media for bacterial growth was from Carl Roth (Karlsruhe, Germany). Chromatography media and columns were produced by GE Healthcare (Little Chalfont, UK). All other chemicals were purchased from Sigma (if not stated otherwise) and were of the highest available purity.

### Cloning and sequencing of recombinant H. pylori AdSS

Genomic DNA of *H. pylori* strains ATCC 26695 was obtained from the collection of the Department of Bacterial Genetics, Institute of Microbiology, Faculty of Biology, University of Warsaw. The PCR fragment corresponding to the *H. pylori purA* gene (gene coding for AdSS protein, GenBank ID: AAD07324.1) was isolated from genomic DNA and amplified using the Phusion High-Fidelity PCR kit (New England BioLabs, Ipswich, MA) with a set of specific DNA primers for both the 5' and the 3' end of the gene. Both DNA primers contained additional flanking regions for introducing *Nde*I and *Xho*I restriction sites at the *purA* gene’s 5' and 3' ends, respectively. Primer *Nde*I-*purA*: 5′-GCGTTGATCATATGGCAGATGTCGTTGTGGG-3′, primer *Xho*I-*purA*: 5′-GGAGGATCTCGAGTCATAGAAAAATCGTGTCTTCTCTTTCAGG-3′. The reaction conditions for PCR amplification were as follows: 30 s hot start at 98 °C followed by 30 cycles of (1) denaturation at 98 °C for 10 s, (2) annealing at 60 °C for 30 s, and (3) elongation at 72 °C for 1 min. The final elongation step was performed at 72 °C for 10 min. The *purA* gene fragment (∼1.2 kb) obtained by PCR was purified from a 1% agarose gel and ligated into the *Nde*I and *Xho*I restricted pET21b expression vector (Invitrogen, Carlsbad, CA). The resulting plasmid, pET21b-*HPpurA*, was transformed into *E. coli* strain XL-10 Gold (Agilent Biotechnology, Santa Clara, CA) for multiplication. After plasmid purification from the bacteria (using GenElute Plasmid Miniprep Kit, Sigma-Aldrich, St. Louis, MO), the DNA sequence of the *H. pylori purA* gene was confirmed by sequence analysis (Macrogen Europe, Amsterdam, Netherlands).

### Expression of recombinant H. pylori AdSS

pET21b*-HPpurA* was subcloned by electroporation into *E. coli* cell strain BL21-CodonPlus(DE3)-RIL (Agilent Biotechnology, Santa Clara, CA) which was used for expression of the recombinant *H. pylori* AdSS. Enzyme expression was performed in the following way. A 10 mL sample of overnight *E. coli* culture was added to 500 ml of LB medium (Carl Roth, Karlsruhe, Germany) containing 100 mg/mL of ampicillin. The cells were grown at 37 °C and 220 rpm to OD_600_ = 0.6 and expression was induced with 0.5 mM IPTG (Isopropyl β-D-1-thiogalactopyranoside). The cells were additionally grown for 4 h at 37 °C and 220 rpm, and harvested at 5000×*g*, at 4 °C.

### Purification of recombinant H. pylori AdSS

Purification was performed at +4 °C, in three chromatographic steps, with a 10 min centrifugation run at 10,000×*g* and +4 °C prior to loading the sample on each column.

Cells were lysed by incubation on ice for 30 min with 1 mg/mL lysozyme in 50 mM phosphate buffer pH 6.5 containing 2 mM EDTA (ethylenediaminetetraacetic acid), 0.1 mM PMSF (phenylmethylsulfonyl fluoride) and 1 mM DTT (dithiothreitol). This was followed by cell sonication for 10 pulses of 30 s each, on ice. Finally, DNaseI was added to the concentration of 3.6 µg/mL and the sample incubated for 10 min at room temperature (∼25 °C) with shaking. Cell debris was separated from protein extract by centrifugation for 20 min at 50,000×*g*, at +4 °C. Any remaining DNA in the protein extract was precipitated with 1% streptomycin sulphate and removed by centrifugation for 15 min at 16,000×*g*, at +4 °C.

As a first step in the purification procedure, the protein extract thus prepared was loaded onto cation-exchange chromatography. SP-Sepharose FF was used (column dimensions 1.6 × 27.2 cm, i.e. 55 mL) with a flow rate of 75 mL/h and fractions of 5.0 mL were collected. The column was equilibrated in 50 mM phosphate buffer pH 6.5 containing 2 mM EDTA and 1 mM DTT. As it was detected that AdSS was not bound to SP-Sepharose FF under given conditions, all bound proteins were eluted with 1 M NaCl in starting buffer.

Combined fractions containing AdSS were concentrated through a PM-30 membrane (Merck, Darmstadt, Germany) in an Amicon stirred cell and loaded onto a size-exclusion chromatography (SEC) column (1.6 × 94.5 cm, i.e. 190 mL) of Sephacryl S-200 HR (loaded sample volume equalled 1.5% of column volume). Flow rate was maintained at 11 mL/h and 2.3 mL fractions were collected. In this step 50 mM phosphate buffer pH 6.5 containing 150 mM NaCl, 2 mM EDTA and 1 mM DTT was used.

AdSS-containing fractions were pulled, concentrated through a PM-30 membrane and transferred to 50 mM Tris–HCl buffer pH 8.5 containing 1 mM DTT (buffer A), by means of SEC on a PD-10 column (GE Healthcare). The sample thus prepared was loaded onto the anion exchange chromatography column MonoQ 5/50 GL on ÅKTA protein purification system (GE Healthcare). The column was equilibrated in buffer A and elution was performed by gradually increasing the concentration of NaCl (by mixing buffer A with buffer B containing 1 M NaCl in buffer A): 0–30% B in 20 mL and 30–100% B in 5 mL. AdSS was found to elute at ∼85 mM NaCl. During this chromatography, flow rate was maintained at 60 mL/h and 1.0 mL fractions were collected.

The final sample of AdSS for use in further experiments was prepared by concentrating the enzyme through a 10 kDa MWCO Hydrosart membrane in the Vivaspin2 device (Sartorius, Göttingen, Germany) and transferring it to 20 mM HEPES-NaOH buffer pH 6.5 containing 2 mM TCEP (tris(2-carboxyethyl)phosphine hydrochloride) by dialysis in the Tube-O-DIALYZER Micro device (GBiosciences, St. Louis, MO).

The purification was monitored by electrophoresis under denaturing conditions (as described below) and a protein concentration assay following the method of Bradford^27^ with bovine serum albumin as a standard.

SEC on a Sephacryl S-200 HR column (under the conditions described) was also used to estimate the molecular mass/oligomeric state of *H. pylori* AdSS. The column was calibrated with several proteins from the Gel Filtration Calibration Kit (GE Healthcare) – chymotrypsinogen A (25 kDa), bovine serum albumin (67 kDa), aldolase (158 kDa), and catalase (232 kDa), together with Blue Dextran. Ultraviolet absorption detection at 280 nm was used for determining the elution volume of the marker proteins.

### Electrophoresis under denaturing conditions

To follow the purification of *H. pylori* AdSS and to estimate the final preparation purity and monomer molecular mass, horizontal SDS-PAGE was performed on a PhastSystem apparatus (GE Healthcare) following the manufacturer's procedures. PhastGel 12.5% plates and SDS buffer strips were used, both from the same producer, as well as the protein low molecular weight (LMW) markers (14.4–93.0 kDa). Vertical SDS-PAGE was performed in a Mini-PROTEAN Tetra Cell (Bio-Rad, Hercules, CA), according to the manufacturer’s instructions. Gels (12.5%) for this system were casted in-house, according to the manufacturer’s instructions. LMW markers were also used in this system.

Prior to loading, samples were mixed with treatment buffer (PhastSystem – 20 mM Tris–HCl pH 8.0, 2 mM EDTA, 5% SDS, 10% 2-mercaptoethanol; Mini-PROTEAN system – 0.125 M Tris–HCl pH 6.8, 4% SDS, 20% glycerol, 2% 2-mercaptoethanol) in ratio 1:1 (v/v) and heated for 5 min at 95 °C. Gels were stained with Coomassie Brilliant Blue R-250 (GE Healthcare) according to the manufacturer's instructions.

### Enzyme activity assays

AdSS catalyses the reaction shown in the Introduction, utilising three substrates: IMP, GTP, and Asp. The enzyme activity was determined for the purified protein at pH 7.7, in 20 mM HEPES-NaOH buffer at 25 °C. The reaction mixture contained 0.15 mM IMP, 5 mM Asp, 0.06 mM GTP and 1 mM MgCl_2_ in the above-mentioned buffer. The reaction course was monitored spectrophotometrically by adding AdSS (approximately 1 µg) to 1 mL of the reaction mixture and by measuring the change in absorption at 280 nm for three minutes. If nucleosides were omitted from the reaction mixture (control for aspartase activity), the purified sample showed no change in absorbance. During purification, aspartase activity interfered too much with the AdSS assay, and these measurements are not exhibited in the results. The extinction coefficient used to calculate the amount of the products formed was 1.17 × 10^4 ^M^−1 ^cm^−1^ (formation of adenylosuccinate)^6^.

One unit (U) of AdSS enzymatic activity is defined as µmol of adenylosuccinate formed per min at 25 °C. Specific activity is expressed as units per mg of protein (U/mg).

### Analytical ultracentrifugation

The oligomeric state of AdSS was studied by analytical centrifugation with absorbance and interference detection, and with the sedimentation velocity method. Optima XL-I ultracentrifuge (Beckman-Coulter Inc., Indianapolis, IN), An-50Ti and An-60Ti analytical rotors, and double-sector 1.2-cm cells with Epon-charcoal centrepieces with sapphire or quartz windows were used. The partial specific volume of *H. pylori* AdSS from its amino acid composition and densities and viscosities of buffers were calculated using the Sednterp programme^28^.

Experiments were conducted at 50,000 rpm in 20 mM HEPES-NaOH pH 6.8 + 1 mM TCEP, at 4 °C or at 20 °C and in 50 mM Tris–HCl +2 mM EDTA +1 mM DTT pH = 8.5 at 20 °C (pH = 9.09 at 4 °C). The protein samples (390 µL) had a concentration in the range 0.15–1.01 mg/mL (3.3–22.1 µM of enzyme subunits). The extinction coefficient ɛ_280nm_ = 38,850 M^−1^ cm^−1^ according to ProtParam^29^ was used to calculate the enzyme concentration.

The following ligands were used to get various binary, ternary, and quaternary complexes of the enzyme: IMP (0.1 mM and 3.0 mM), GDP (5.0 mM), GTP (4.0 mM), hadacidin (1 and 5.5 mM) and MgSO_4_ (3.25 mM). Presence of GDP and GTP in a concentration necessary to obtain complexes with AdSS causes the absorbance to exceed the allowed limit. Therefore, interference detection was used for all samples, while absorbance detection at 280 nm was utilised only in three cases: for the apo enzyme, the binary complex with 0.1 mM IMP, and the ternary complex with 0.1 mM IMP and 1 mM hadacidin.

Radial scans of the absorption and interference profiles in the cell were measured at 5- or 7-min intervals on the same protein samples. Analysis of the ultracentrifugation data was done by the Sedfit programme using the continuous *c*(*S*) distribution model (www.analyticalultracentrifugation.com/)^30^. Both the sedimentation coefficient *s* and the standard sedimentation coefficient *s*_20,w_ were calculated, and based on them the molecular mass of the species present in the solution was determined, assuming that they have the same friction ratio (also obtained as a parameter in the Sedfit programme). Results shown in the text and in the Tables 1 and 2 are arithmetic means with standard error.

**Table 1.** Distribution of *Helicobacter pylori* AdSS between dimeric and monomeric forms, sedimentation coefficients, and molecular masses of these forms, obtained from the analytical ultracentrifugation studies of the apo enzyme, its binary complex with IMP and ternary complex with IMP and hadacidin.

	Number of experiments	Detection	Monomer			Dimer		
			s^0^_w,20_ [S]	MW^a^ [kDa]	*c(s)* [%]	s^0^_w,20_ [S]	MW^a^ [kDa]	*c(s)* [%]
pH 6.8, 4 °C and 20 °C^b^								
AdSS (4.4 or 14.9 µM)	4(7^a^)	A_280 nm_	3.35–3.70	44.1 ± 1.8	2.0–16.9	5.27–5.82	88.0 ± 2.0	83.1–98.0
AdSS (5.1 µM)	1	Interference	3.73	61.1	8.7	5.70	115.5	91.3
AdSS (4.4 or 14.9 µM) IMP (0.1 mM)	3	A_280 nm_	3.10–3.82	42.8 ± 5.0	2.8–3.4	5.75–5.91	92.4 ± 3.8	96.6–97.2
AdSS (4.4 or 14.9 µM) IMP (0.1 mM) Hadacidin (1 mM)	3	A_280 nm_	2.70–3.53	35.0 ± 3.9	3.4–5.7	5.75–5.91	89.4 ± 0.7	94.3–96.6
pH 9.1, 4 °C^c^								
AdSS (5.0 µM)	1	A_280 nm_	3.41	45.3	28.7	5.57	94.5	71.3
AdSS (13.4 µM)	1	A_280 nm_	3.54	49.0	28.0	5.45	93.8	72.0
AdSS (22.1 µM)	1	A_280 nm_	3.59	48.6	29.1	5.27	86.7	70.9

The sedimentation velocity method and absorbance at 280 nm and/or interference detection were used.

a To determine MW of the apo enzyme results in TRIS–HCl pH 9.1, 4 °C were also included.

b Experiments at 20 mM HEPES-NaOH pH 6.8 + 1 mM TCEP, enzyme activity 1.19 ± 0.10 U/mg.

c Experiments at 50 mM TRIS–HCl pH 9.1 (at 4 °C) + 2 mM EDTA +1 mM DTT, enzyme activity 0.75 U/mg.

**Table 2.** Distribution of AdSS between monomeric and dimeric forms observed in the analytical ultracentrifugation studies for various enzyme complexes.

pH 6.8, 20 °C^a^	Monomer	Dimer
	c(s)^b^ [%]	c(s)^b^ [%]
AdSS + GTP + IMP + MgSO_4_ (5.1 µM + 3.0 mM +3.0 mM +3.25 mM)	10.2	89.8
AdSS + GDP + IMP (5.1 µM + 6.0 mM +3.0 mM)	7.3	92.7
AdSS + GDP + IMP + MgSO_4_ (5.1 µM + 6.0 mM +3.0 mM +3.25 mM)	14.9	85.1
AdSS + IMP (5.1 µM + 3.0 mM)	11.3	88.7
AdSS + GDP + IMP + MgSO_4_+Hadacidin (5.1 µM + 6.0 mM +3.0 mM +3.25 mM +5.5 mM)	14.0	86.0
AdSS + GTP + IMP + MgSO_4_+Hadacidin (5.1 µM + 3.0 mM +3.0 mM +3.25 mM +5.5 mM)	14.4	85.6

The sedimentation velocity method with interference detection was used.

a Experiments at 20 mM HEPES-NaOH pH 6.8 + 1 mM TCEP, enzyme activity 1.02 U/mg.

b *c*(*S*) [%] under assumption that dimer and monomer are the only species present in the sample.

### Temperature and pH effects on H. pylori AdSS stability and activity

In all temperature and pH dependence studies, the AdSS activity assay was used, as described above. The *H. pylori* AdSS concentration in all reaction mixtures was 1 µg/mL. All assays were done in triplicate.

In experiments determining the effect of temperature on enzyme activity, the reaction was performed at various temperatures ranging from 25 to 70 °C, after 5 min of incubation of the reaction mixture at a given temperature. The thermal stability of *H. pylori* AdSS was determined by incubating the enzyme (0.1 mg/mL) in 20 mM HEPES-NaOH buffer pH 7.7 for 1 h at various temperatures in the range 25–50 °C. And 10 µL of the incubation mixture was used for each activity measurement.

The reaction mixtures for testing the pH influence on the activity of *H. pylori* AdSS contained Britton–Robinson universal buffer (BR buffer, a mixture of 40 mM boric acid, 40 mM phosphoric acid, and 40 mM acetic acid, titrated with 1 M NaOH)^31^ with pH in the range from 5.0 to 9.0, instead of the HEPES-NaOH buffer pH 7.7. The same molar extinction coefficient as above (*ε* = 1.17 × 10^4^ M^−1^ cm^−1^) was used in the calculation of enzyme activity, as the spectrum of adenylosuccinate was shown not to change in this pH range^32^, which we also experimentally checked. The effect of pH on *H. pylori* AdSS stability was tested by incubating the enzyme (0.1 mg/mL) in BR buffer at different pH values (5.0–9.5) for 1 h at 25 °C. Then, 10 µL of incubation mixture was used for each activity measurement under standard conditions (HEPES-NaOH buffer pH 7.7, 25 °C).

### Determination of kinetic constants

For the determination of kinetic constants, the concentration of one substrate was varied at fixed saturating concentrations of the other two substrates (0.06 mM for GTP, 0.15 mM for IMP and 5 mM for Asp). The concentration of GTP was varied between 0.6 and 120 µM, of IMP between 0.9 and 225 µM, and of Asp between 0.01 and 7.5 mM. The data were analysed by fits of the Michaelis–Menten equation (Equation (1)) using GraphPad Prism version 7.03 (GraphPad Software, Inc., San Diego, CA).

(1) $$V=V_{\text{max}}\left[S\right]/\left(K_{m}+\;\left[S\right]\right)$$

where *V* and *V*_max_ are the initial velocity and maximum velocity, respectively, *K*_m_ is the Michaelis constant and [*S*] is the variable substrate concentration.

In order to determine the *K*_i_ value of hadacidin, concentrations of GTP and IMP were maintained at saturating levels (0.06 and 0.15 mM, respectively), while initial concentrations of Asp and hadacidin were varied from 0.01 to 1 mM and from 0.3 to 4 µM, respectively. On the other hand, in order to determine the *K*_i_ value of adenylosuccinate, concentrations of GTP and Asp were maintained at saturating levels (0.06 and 5 mM, respectively), while initial concentrations of IMP and adenylosuccinate were varied from 7.5 to 225 µM and from 10 to 30 µM, respectively. The data were analysed by fits of the competitive inhibition model (Equation (2)) using GraphPad Prism version 7.03.

(2) $$V=V_{\text{max}}\left[S\right]/\left\{K_{m}\left(1+\left[I\right]/K_{i}\right)+\left[S\right]\right\}$$

where *V* and *V*_max_ are the initial velocity and maximum velocity, respectively, *K*_m_ is the Michaelis constant, *K*_i_ is the inhibition constant and [*S*] is the variable substrate concentration.

## Results and discussion

There are eight reviewed and over 400 un-reviewed AdSS protein sequences from *Helicobacter* species in the UniProt database (July 2018). However, none has been studied in detail, yet.

### Enzyme purification

**Figure 1.**
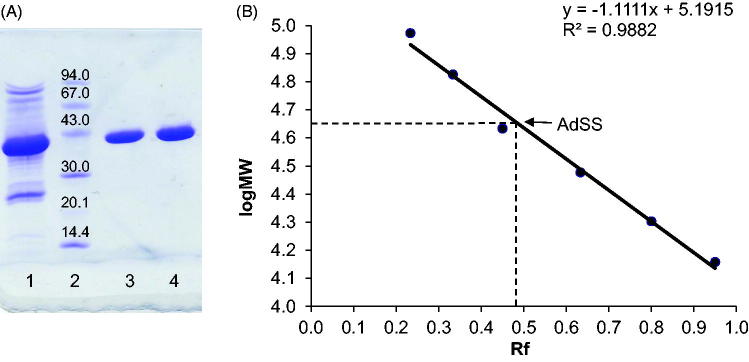
(A) SDS-PAGE of purified *H. pylori* AdSS, lane 1 – pulled fractions after SEC on Sephacryl S-200 HR, lane 2 – LMW protein markers (molecular masses in kDa given in the figure), lanes 3 & 4 – purified AdSS. (B) Calibration curve for the given gel, Rf is ratio of migration distance of the protein and migration distance of the dye front.

AdSS from the pathogen *H. pylori* was purified using a three-step procedure: cation exchange, size exclusion, and anion exchange chromatography. The enzyme appeared homogeneous on SDS-PAGE (Figure 1) and its molecular mass estimated from the SDS gel (45.0 kDa) corresponds very well with the theoretical value (for a monomer) of 45,742.72 Da (as calculated by the ProtParam tool^29^ on the ExPASy server). The molecular mass estimated from SEC on the Sephacryl S-200 HR column is 96.5 kDa (Supplementary Figure S1), which indicates the existence of an enzyme dimer under given conditions (see Materials and methods section).

According to the available literature, bacterial AdSS enzymes are anionic and bind to anion exchangers, same as acidic mammalian isozymes^1^. Most of them have a monomer molecular mass of ∼50 kDa and require dimerisation for achieving full enzymatic activity^1^. The molecular mass of a *H. pylori* AdSS monomer fits within the range of the so far characterised bacterial AdSS enzymes. The elution volume from the SEC column indicates that relatively high protein concentration (usually ∼15 mg/mL of total protein concentration in the sample loaded onto SEC) combined with 0.15 M NaCl favour the presence of *H. pylori* AdSS dimers. A shift towards monomers at higher ionic strength (200 mM phosphate buffer) was observed for *P. falciparum* AdSS^33^, while a shift towards dimers at higher protein concentrations and with the presence of ligands was demonstrated for *E. coli* AdSS^4^.

The predicted pI for *H. pylori* AdSS (7.53, as calculated by the ProtParam tool^29^ on the ExPASy server) is significantly higher than the one predicted for other bacterial AdSS enzymes (7.53 compared to 5–6 for AdSS from other bacteria). Binding to an anion exchanger in the final purification step indicates the total protein charge to be ≤7.5. Interestingly, the pI of *H. pylori* AdSS is very similar to the pI of *P. falciparum* AdSS (pI 7.54). These parasitic protozoa also lack the *de novo* purine synthesis pathway, and similarly to *H. pylori* rely completely on the purine salvage pathway for their purine nucleotide requirements^33^.

### Determining the enzyme’s oligomeric state in the absence and presence of ligands

Monomeric (inactive) and dimeric (active) forms of *E. coli* AdSS are in dynamic equilibrium, and the enzyme exists solely in a dimeric form either when the enzyme concentration is high (several mg/mL) or when ligands are present. The *K*_d_ of a dimer falls from 10 µM in the absence of ligands to a value near zero in their presence^4^^,^^34^. To check if AdSS from *H. pylori* also shows such a behaviour, the oligomeric form of the apo enzyme, and its various binary, ternary and quaternary complexes were studied by analytical centrifugation using the sedimentation velocity method.

**Figure 2.**
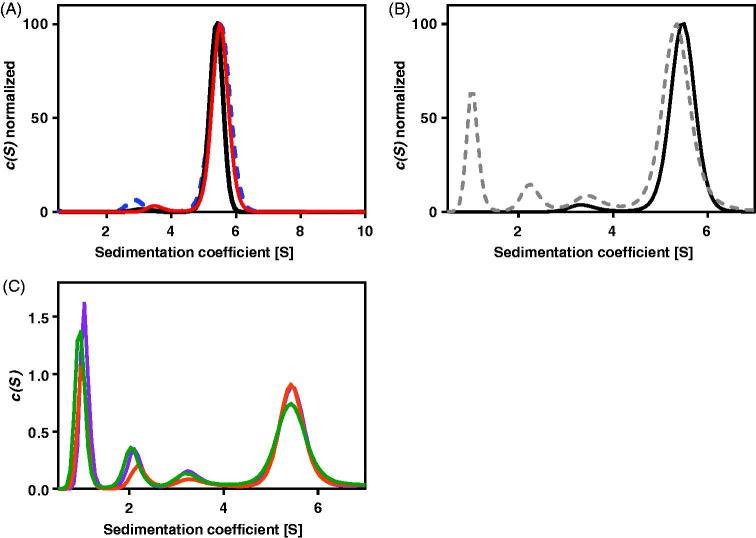
The absorption at 280 nm, A_280nm_(s), and the interference(s) profiles obtained from fitting the continuous c(s) distribution model to the sedimentation velocity data acquired for *Helicobacter pylori* AdSS at 20 mM HEPES-NaOH pH 6.8 + 1 mM TCEP, at 20 °C. (A) A_280 nm_(s) profile for the 4.4 µM of the apo AdSS (black); 4.4 µM AdSS in a binary complex with 0.1 mM IMP (red); and 3.3 µM AdSS in a ternary complex with 0.1 mM IMP and 1 mM hadacidin (blue dashed line) (data normalised). (B) A_280 nm_(s) (black) and interference(s) (grey dashed line) profiles for the apo enzyme, 5.1 µM (data normalised). (C) Interference(s) profiles for the 5.1 µM AdSS in a binary complex with 3.0 mM IMP (orange); ternary complex with 3 mM IMP and 6 mM GDP, in the presence of 3.3 mM MgSO_4_ (violet); and quaternary complex with 3.0 mM IMP, 6.0 mM GDP and 5.5 mM hadacidin, in the presence of 3.3 mM MgSO_4_ (green). Two species with sedimentation coefficients of about 1S and 2S, corresponding to a molecular mass of about 10 and 30 kDa, as observed only in the interference profiles, not in the A_280nm_ profiles (see data on panel B), are therefore probably non-protein contaminants, which is additionally confirmed by the single band observed on the SDS electrophoresis gel (see Figure 1).

**Figure 3.**
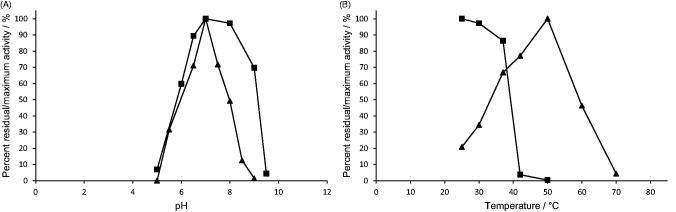
Effect of A) pH, and B) temperature on *H. pylori* AdSS stability (–▪–) and activity (–▲–). The exact conditions are described in Materials and methods section.

At pH 6.8 where AdSS has the maximum of activity and stability (Figure 3), ultracentrifugation experiments (Figure 2), conducted for 0.2 mg/mL (4.4 µM) and 0.68 mg/mL (14.9 µM) AdSS, at 4 °C and at 20 °C, with absorption detection at 280 nm, show that the majority of the sample (from 83.1 to 98.0%, see Table 1) constitutes a species with a sedimentation coefficient s^0^_20,w_ in the range 5.70–5.82 S and molecular mass MW = 88.0 ± 2.0 kDa, hence corresponding to a dimer (the theoretical mass of the monomer from its sequence is 45,742.72 Da). The rest of the protein is in the monomeric form with MW = 44.1 ± 1.8 kDa. However, a very similar amount of the monomer (see Table 1) is observed also in the presence of ligands, 0.1 mM IMP alone and with addition of 1 mM hadacidin (competitive inhibitor of aspartic acid, see Supplementary Table S1 and section below). This suggests that, in contrast to *E. coli* AdSS^4^^,^^35^, monomer and dimer of the *H. pylori* counterpart may not be in a dynamic equilibrium and binding of ligands by this enzyme may not push the equilibrium towards the dimer.

The presence of a higher concentration of IMP, as well as GTP or GDP, results in absorption exceeding the allowed limit, therefore the following experiments were done with interference detection only, which enables the use of high concentrations of all ligands, including those absorbing in the near UV region. Comparison of both detection methods for the apo enzyme (see Figure 2(B) and Table 1) shows that they both give similar results regarding the monomer/dimer ratio, thus justifying the use of the interference detection method to determine the distribution of AdSS complexes between dimeric and monomeric forms.

Interference detection experiments were therefore conducted for various binary, ternary and quaternary AdSS complexes and the results obtained are shown in Figure 2(C) and Table 2. These data indicate that none of the ligand combinations used in these studies, despite the high ligand concentrations utilised, were able to increase significantly the amount of dimers relative to monomers. Hence this proves that *H. pylori* AdSS is a dimer even at concentrations as low as several µM, regardless of the presence of ligands, and that the enzyme subunits indeed are not in a dynamic equilibrium. The data also suggest that the monomer observed in the experiments is most probably an irreversible intermediate on the inactivation pathway of the enzyme. This is further supported by the results of the next experiment, conducted at pH 9.1 (Table 1), hence under conditions where the enzyme is less stable (see Figure 3), which showed an increased amount of the monomeric form (28.0–29.1%), regardless the enzyme concentration of the sample used.

These data suggest that the AdSS form of *H. pylori* is a dimer *in vivo* and its activity is not regulated, like that of the *E. coli* enzyme, by a substrate-induced dimerisation. This seems to be in line with the fact that the *de novo* purine synthesis pathway is missing in *H. pylori* and the purine salvage pathway is the only source of purines for this organism^22^. It seems likely that the enzymes of this pathway are constantly active to meet the purine nucleotide demands of the organism.

### Effect of pH and temperature on enzyme activity and stability

Purified *H. pylori* AdSS was most active under chosen conditions (see Materials and methods section) at pH 7.0 and 50 °C (Figure 3). The enzyme was stable (retaining ≥90% of the initial activity) in the pH range 6.5–8.5 at 25 °C and up to 37 °C at pH 7.0 (Figure 3).

Purified AdSS retained its full activity for 11 days when incubated at 25 °C at a concentration of 0.57 mg/mL, and lost no activity through several cycles of freezing (−80 °C) and thawing.

There is a surprisingly low amount of data on AdSS proteins regarding their stability and optimal catalysis conditions. In the review by Stayton et al.^1^ only a note on pH optimum can be found, stating that AdSS enzymes from various sources have pH optima in the pH range 6.5–7.5. Additionally, we were able to find data for AdSS from *P. falciparum* – pH optimum in the pH range 6.8–7.5^33^. AdSS from yeast *Saccharomyces cerevisiae* was better characterised – optimal activity at 35 °C and pH = 7.0, enzyme stable in the wide pH range 5–10 and up to 37 °C^36^. Temperature optimum is known to be the result of two processes – rise in enzyme activity and protein denaturation with rising temperature^37^. With that in mind, the temperature optimum of *H. pylori* AdSS of 50 °C in comparison with its low temperature stability can be explained by the short time of enzymatic activity measurement (3 min).

*H. pylori* has been evolving alongside humans for hundreds of thousands of years and thus became very well adapted to conditions in the human stomach^24^. Its metabolism is optimised for living in the acid environment of the stomach and for using available nutrients for growth^22^. For example, a characteristic feature of *H. pylori* is the generation of the enzyme urease that catalyses decomposition of urea. As one of the products is ammonia, this reaction results in an increase of pH in the immediate environment. This is one of the key factors of survival of *H. pylori* in an unfavourable environment^14^^,^^20^^,^^38^. In this context, it is understandable that the pH optimum of *H. pylori* AdSS is in the neutral pH range and that the enzyme is stable and active at physiological temperatures maintained within the human body, the host of this pathogen.

### Kinetic characterisation of the enzymatic reaction

Kinetic constants determined for all three substrates of AdSS, IMP, GTP, and Asp are given in Table 3. For each substrate, the curve of initial velocity over substrate concentration follows Michaelis–Menten kinetics (Equation (1)). When comparing Michaelis constants of *H. pylori* AdSS with those of other organisms, it can be observed that they are similar to those of e.g. *E. coli* AdSS (GTP: 10–48 µM, IMP: 20–200 µM, and Asp: 260–350 µM) or mouse basic isozyme (GTP: 12 µM, IMP: 45 µM, and Asp: 140 µM), while e.g. the mouse acidic isozyme (GTP: 15 µM, IMP: 12 µM, and Asp: 950 µM) or *P. falciparum* AdSS (GTP: 18 µM, IMP: 23 µM, and Asp: 1800 µM) have significantly higher values of *K*_m_ for Asp^1^^,^^39^. Turnover number (*k*_cat_) of 1 s^−1^ compares well with *k*_cat_ for *E. coli* and *P. falciparum* AdSS enzymes, however, both mouse enzymes have 4- to 5-fold higher efficacy^39^. This low value of *k*_cat_ makes AdSS one of the least efficient enzymes described in the literature, and for *E. coli* the AdSS reaction is near the rate-limiting step in the bacterium’s growth and reproduction cycle^3^. This is in line with the known fact that purine metabolism is the growth-limiting step for all cells, both prokaryotic and eukaryotic^24^.

**Table 3.** Kinetic parameters for *H. pylori* AdSS, obtained by fitting the Michaelis–Menten equation to the experimental data. Fitting errors of the kinetic parameters obtained are shown in the table.

Variable substrate	Fixed substrates’ concentration (mM)	*K*_m_ (µM)	*V*_max_ (U/mg)
GTP	IMP – 0.15, Asp – 5.0	8.7 ± 0.6	1.418 ± 0.023
IMP	GTP – 0.06, Asp – 5.0	40.1 ± 2.9	1.456 ± 0.036
Asp	GTP – 0.06, IMP – 0.15	125.4 ± 7.7	1.103 ± 0.016

### Enzyme inhibition with hadacidin

**Figure 4.**
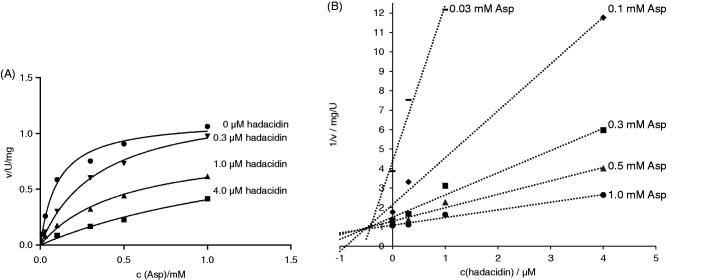
Inhibition of *H. pylori* AdSS by hadacidin, at 25 °C, in 20 mM HEPES-NaOH buffer pH 7.7, with Asp as a variable substrate. (A) Initial velocity (v) vs. substrate concentration, for inhibitor concentrations indicated in the figure. (B) The same data shown on the Dixon plot, i.e. reciprocal of the initial velocity (1/v) vs. inhibitor concentration, for substrate concentrations indicated in the figure.

Hadacidin is a natural antibiotic, the fermentation product of *Penicillium frequentans*^40^. It has been shown to diminish AdSS activity as a competitive inhibitor of aspartic acid^3^. The inhibition constant (*K*_i_) for its activity on *H. pylori* AdSS was determined to be 0.19 ± 0.02 µM. The competitive effect of hadacidin on AdSS activity was confirmed by plots of initial velocity over substrate concentration and Dixon plots (1/v vs. inhibitor concentration), as shown in Figure 4.

The hadacidin inhibition constant of *H. pylori* AdSS is the lowest known so far. Those reported for other enzymes span from 0.3 µM for AdSS from *Aztobacter vinelandii*^41^ to 10.5 µM for the same enzyme from the thermophilic archaeon *Methanocaldococcus jannaschii*^39^. For *E. coli* AdSS values from 1.0 to 4.2 µM were reported^42^^,^^43^, and for *P. falciparum* 5.6 µM^7^. Attempts have been made to improve hadacidin inhibition of AdSS by creating its analogous, with no significant results^44^^,^^45^.

### Enzyme inhibition with adenylosuccinate

**Figure 5.**
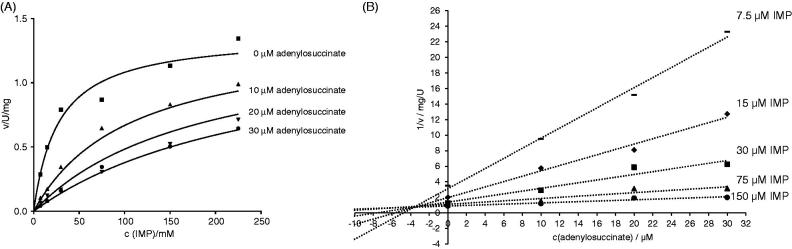
Inhibition of *H. pylori* AdSS by adenylosuccinate, at 25 °C, in 20 mM HEPES-NaOH buffer pH 7.7, with IMP as a variable substrate. (A) Initial velocity (v) vs. substrate concentration, for inhibitor concentrations indicated in the figure. (B) The same data shown on the Dixon plot, i.e. reciprocal of the initial velocity (1/v) vs. inhibitor concentration, for substrate concentrations indicated in the figure.

Adenylosuccinate is the product of the AdSS reaction (see Introduction). The inhibition constant (*K*_i_) for its activity on *H. pylori* AdSS was determined to be 3.5 ± 0.3 µM. The competitive effect of adenylosuccinate on AdSS activity (towards IMP) was confirmed by plots of initial velocity over substrate concentration and Dixon plots (1/v vs. inhibitor concentration), as shown in Figure 5.

AdSS enzymes were shown to be susceptible to product and feedback inhibition. Adenylosuccinate is a strong inhibitor with *K*_i_ in the range 5–30 µM for bacterial enzymes^1^. For the *E. coli* enzyme it was shown that adenylosuccinate inhibits AdSS competitively with respect to IMP with a *K*_i_ of 5 µM^6^. The same behaviour was observed for AdSS enzymes from *A. vinelandii* (*K*_i_ = 2.5 µM)^41^, *M. jannaschii* (*K*_i_ = 9.4 µM)^39^ and *P. falciparum* (*K*_i_ = 29 µM)^7^.

## Conclusions

This article represents the very first description of the biochemical and kinetic properties of AdSS from the bacterium *H. pylori*. Although similar to other characterised bacterial AdSS enzymes (represented by *E. coli* AdSS, protein sequence identity with *H. pylori* AdSS: 43.7%, as calculated by ClustalO^46^ on ExPASy server) in size and kinetic properties, *H. pylori* AdSS also shares attributes (like isoelectric point and oligomeric state) with AdSSs from protozoan parasites (like *P. falciparum*) which also lack the *de novo* purine synthesis pathway. Strong inhibition of *H. pylori* AdSS with hadacidin and adenylosuccinate provides foundation for further work on finding novel inhibiting compounds and solving the 3D-structure of this enzyme in its apo form and in various complexes, which is something we are currently working on. The results presented here on *H. pylori* AdSS, along with future findings, give hope of finding novel antibiotics against this dangerous pathogen.

## Supplementary Material

Supplemental Material

## References

[1] Stayton MM, Rudolph FB, Fromm HJ Regulation, genetics, and properties of adenylosuccinate synthetase: a review. Curr Top Cell Regul 1983;22:103–41. 6347525 10.1016/b978-0-12-152822-5.50008-7

[2] Matsuda Y, Ogawa H, Fukutome S, et al. Adenylosuccinate synthetase in rat liver: the existence of two types and their regulatory roles. Biochem Biophys Res Commun 1977;78:766–71. 71897 10.1016/0006-291x(77)90245-5

[3] Honzatko RB, Stayton MM, Fromm HJ, Adenylosuccinate synthetase: recent developments In: Purich DL, editor. Advances in enzymology and related areas of molecular biology. Hoboken (NJ): John Wiley and Sons Inc; 1999 57–102 p. 10218106

[4] Wang W, Gorrell A, Honzatko R, Fromm HJ A study of *Escherichia coli* adenylosuccinate synthetase association states and the interface residues of the homodimer. J Biol Chem 1997;272:7078–84. 9054400 10.1074/jbc.272.11.7078

[5] Honzatko RB, Fromm HJ Structure-function studies of adenylosuccinate synthetase from *Escherichia coli*. Arch Biochem Biophys 1999;370:1–8. 10496970 10.1006/abbi.1999.1383

[6] Rudolph FB, Fromm HJ Initial rate studies of adenylosuccinate synthetase with product and competitive inhibitors. J Biol Chem 1969;244:3832–9. 4896485

[7] Raman J, Mehrotra S, Anand RP, Balaram H Unique kinetic mechanism of *Plasmodium falciparum* adenylosuccinate synthetase. Mol Biochem Parasitol 2004;138:1–8. 15500910 10.1016/j.molbiopara.2004.06.013

[8] Wyngaarden JB, Greenland RA The inhibition of succinoadenylate kinosynthetase of *Escherichia coli* by adenosine and guanosine 5′-monophosphates. J Biol Chem 1963;238:1054–7. 14002128

[9] Stayton MM, Fromm HJ Guanosine 5′-diphosphate-3′-diphosphate inhibition of adenylosuccinate synthetase. J Biol Chem 1979;8:2579–81. 372184

[10] Hou Z, Cashel M, Fromm HJ, Honzatko RB Effectors of the stringent response target the active site of *Escherichia coli* adenylosuccinate synthetase. J Biol Chem 1999;274:17505–10. 10364182 10.1074/jbc.274.25.17505

[11] Hanessian S, Lu PP, Sancéau JY, et al. An enzyme-bound bisubstrate hybrid inhibitor of adenylosuccinate synthetase. Angew Chem Int Ed Engl 1999;38:3159–62. 10556888 10.1002/(sici)1521-3773(19991102)38:21<3159::aid-anie3159>3.0.co;2-2

[12] Iancu CV, Zhou Y, Borza T, et al. Cavitation as a mechanism of substrate discrimination by adenylosuccinate synthetases. Biochemistry 2006;45:11703–11. 16981730 10.1021/bi0607498 PMC4869520

[13] Marshall BJ, Warren JR Unidentified curved bacilli in the stomach of patients with gastritis and peptic ulceration. Lancet 1984;1:1311–5. 6145023 10.1016/s0140-6736(84)91816-6

[14] Talebi Bezmin Abadi A Strategies used by *Helicobacter pylori* to establish persistent infection. World J Gastroenterol 2017;23:2870–82. 28522905 10.3748/wjg.v23.i16.2870 PMC5413782

[15] Miftahussurur M, Yamaoka Y, Graham DY *Helicobacter pylori* as an oncogenic pathogen, revisited. Expert Rev Mol Med 2017;19:1–11. 10.1017/erm.2017.4 PMC6905048 28322182

[16] Zamani M, Ebrahimtabar F, Zamani V, et al. Systematic review with meta-analysis: the worldwide prevalence of *Helicobacter pylori* infection. Aliment Pharmacol Ther 2018;47:868–76. 29430669 10.1111/apt.14561

[17] Peleteiro B, Bastos A, Ferro A, Lunet N Prevalence of *Helicobacter pylori* infection worldwide: a systematic review of studies with national coverage. Dig Dis Sci 2014;59:1698–709. 24563236 10.1007/s10620-014-3063-0

[18] Suzuki H, Mori H World trends for *H. pylori* eradication therapy and gastric cancer prevention strategy by *H. pylori* test-and-treat. J Gastroenterol 2018;53:354–61. 29138921 10.1007/s00535-017-1407-1 PMC5847180

[19] Thung I, Aramin H, Vavinskaya V, et al. Review article: the global emergence of *Helicobacter pylori* antibiotic resistance. Aliment Pharmacol Ther 2016;43:514–33. 26694080 10.1111/apt.13497 PMC5064663

[20] Tomb JF, White O, Kerlavage AR, et al. The complete genome sequence of the gastric pathogen *Helicobacter pylori*. Nature 1997;389:539–47. 9252185 10.1038/41483

[21] Doig P, de Jonge BL, Alm RA, et al. *Helicobacter pylori* physiology predicted from genomic comparison of two strains. Microbiol Mol Biol Rev 1999;63:675–707. 10477312 10.1128/mmbr.63.3.675-707.1999 PMC103750

[22] Hazell SL, Mendz GL How *Helicobacter pylori* works: an overview of the metabolism of *Helicobacter pylori*. Helicobacter 1997;2:1–12. 9432315 10.1111/j.1523-5378.1997.tb00050.x

[23] el Kouni MH Potential chemotherapeutic targets in the purine metabolism of parasites. Pharmacol Ther 2003;99:283–309. 12951162 10.1016/s0163-7258(03)00071-8

[24] Liechti G, Goldberg JB *Helicobacter pylori* relies primarily on the purine salvage pathway for purine nucleotide biosynthesis. J Bacteriol 2012;194:839–54. 22194455 10.1128/JB.05757-11 PMC3272961

[25] Narczyk M, Bertoša B, Papa L, et al *Helicobacter pylori* purine nucleoside phosphorylase shows new distribution patterns of open and closed active site conformations and unusual biochemical features. FEBS J 2018;285:1305–25. 29430816 10.1111/febs.14403

[26] Štefanić Z, Mikleušević G, Luić M, et al. Structural characterization of purine nucleoside phosphorylase from human pathogen *Helicobacter pylori*. Int J Biol Macromol 2017;101:518–26. 28336275 10.1016/j.ijbiomac.2017.03.101

[27] Bradford MM A rapid and sensitive method for the quantitation of microgram quantities of protein utilizing the principle of protein-dye binding. Anal Biochem 1976;72:248–54. 942051 10.1016/0003-2697(76)90527-3

[28] Hayes D, Laue T, Philo J, Program SEDNTERP: sedimentation interpretation program. Thousand Oaks (CA): Alliance Protein Laboratories; 1995.

[29] Gasteiger E, Hoogland C, Gattiker A, et al. Protein identification and analysis tools on the ExPASy server In: Walker J, editor. The proteomics protocols handbook. New York (NY): Humana Press; 2005 571–607 p.

[30] Schuck P Size-distribution analysis of macromolecules by sedimentation velocity ultracentrifugation and lamm equation modeling. Biophys J 2000;78:1606–19. 10692345 10.1016/S0006-3495(00)76713-0 PMC1300758

[31] Britton HTK, Robinson RA Universal buffer solutions and the dissociation constant of veronal. J Chem Soc 1931;0:1456–62.

[32] Carter CE, Cohen LH The preparation and properties of adenylosuccinase and adenylosuccinic acid. J Biol Chem 1956;222:17–30. 13366975

[33] Jayalakshmi R, Sumathy K, Balaram H Purification and characterization of recombinant *Plasmodium falciparum* adenylosuccinate synthetase expressed in *Escherichia coli*. Protein Expr Purif 2002;25:65–72. 12071700 10.1006/prep.2001.1610

[34] Kang C, Kim S, Fromm HJ Subunit complementation of *Escherichia coli* adenylosuccinate synthetase. J Biol Chem 1996;271:29722–8. 8939906 10.1074/jbc.271.47.29722

[35] Hou Z, Wang W, Fromm HJ, Honzatko RB IMP alone organizes the active site of adenylosuccinate synthetase from *Escherichia coli*. J Biol Chem 2002;277:5970–6. 11741996 10.1074/jbc.M109561200

[36] Ryzhova TA, Andreichuk YV, Domkin VD Adenylosuccinate synthetase of the yeast *Saccharomyces cerevisiae*: purification and properties. Biochem (Mosc) 1998;63:650–6. 9668204

[37] Barton JS Denaturation at the optimum temperature. Biochem Educ 1979;7:13–14.

[38] Marshall BJ, Barret LJ, Prakash C, et al. Urea protects *Helicobacter* (*Campylobacter*) *pylori* from the bactericidal effect of acid. Gastroenterology 1990;99:697–702. 2379775 10.1016/0016-5085(90)90957-3

[39] Mehrotra S, Balaram H Kinetic characterization of adenylosuccinate synthetase from the thermophilic archaea *Methanocaldococcus jannaschii*. Biochemistry 2007;46:12821–32. 17929831 10.1021/bi701009y

[40] Kaczka EA, Gitterman CO, Dulaney EL, Folkers K Hadacidin, a new growth-inhibitory substance in human tumor systems. Biochemistry 1962;1:340–3. 14452968 10.1021/bi00908a022

[41] Markham GD, Reed GH Adenylosuccinate synthetase from *Azotobacter vinelandii*: purification, properties and steady-state kinetics. Arch Biochem Biophys 1977;184:24–35. 21629 10.1016/0003-9861(77)90322-8

[42] Poland BW, Lee SF, Subramanian MV, et al. Refined crystal structure of adenylosuccinate synthetase from *Escherichia coli* complexed with hydantocidin 5′-phosphate, GDP, HPO_4_^2-^, Mg^2+^, and hadacidin. Biochemistry 1996;35:15753–9. 8961938 10.1021/bi961758r

[43] Rudolph FB Purification, properties, and kinetics of adenylosuccinate synthetase from *Escherichia coli* [PhD thesis]. Ames (IA): Iowa State University; 1971.

[44] Jahngen EGE, Rossomando EF Adenylosuccinate synthetase from *Dictyostelium discoideum*: effects of hadacidin analogs and binding of [14C]hadacidin. Arch Biochem Biophys 1984;229:145–54. 6703692 10.1016/0003-9861(84)90139-5

[45] Tibrewal N, Elliott GI Evaluation of hadacidin analogues. Bioorg Med Chem Lett 2011;21:517–9. 21129960 10.1016/j.bmcl.2010.10.088

[46] Sievers F, Wilm A, Dineen DG, et al. Fast, scalable generation of high-quality protein multiple sequence alignments using Clustal Omega. Mol Syst Biol 2011;7:539–44. 21988835 10.1038/msb.2011.75 PMC3261699

