# Supplementary material for: In the quest for new targets for pathogen eradication: the adenylosuccinate synthetase from the bacterium Helicobacter pylori
Source: J Enzyme Inhib Med Chem. 2018 Sep 7;33(1):1405–14. doi: 10.1080/14756366.2018.1506773 (PMC6136348; doi:10.1080/14756366.2018.1506773)
Supplement: Supplemental Material [file IENZ_A_1506773_SM5516.pdf]

Table 1Suppl. Inhibitors of *E. coli* AdSS

**Inhibitor**	**Inhibition constant, K_i (μM)**	**Reference**
AMP	95	8
Adenylosuccinate	270<br>5	8<br>6
GDP	23<br>12	8<br>6
GMP	74	8
ppGpp	50	9
ppG2':3'p	0.1	10
6-Mercaptopurine riboside 5'-phosphate	10	6
β,γ-5'-Guanylyl methylene diphosphonate	80	6
hydantocidin 5'-phosphate (HMP)	0.022<br>(IC_{50}) 0.675	3<br>11
Hadacidin	0.49<br>(IC_{50}) 3.5	12<br>11
HMP-hadacidin hybrid inhibitor, (*S*)-isomer	(IC_{50}) 0.043	11
HMP-hadacidin hybrid inhibitor, (*R*)-isomer	(IC_{50}) 0.665	11
Succinate	7500<br>890	6<br>47
Maleate	3100	47

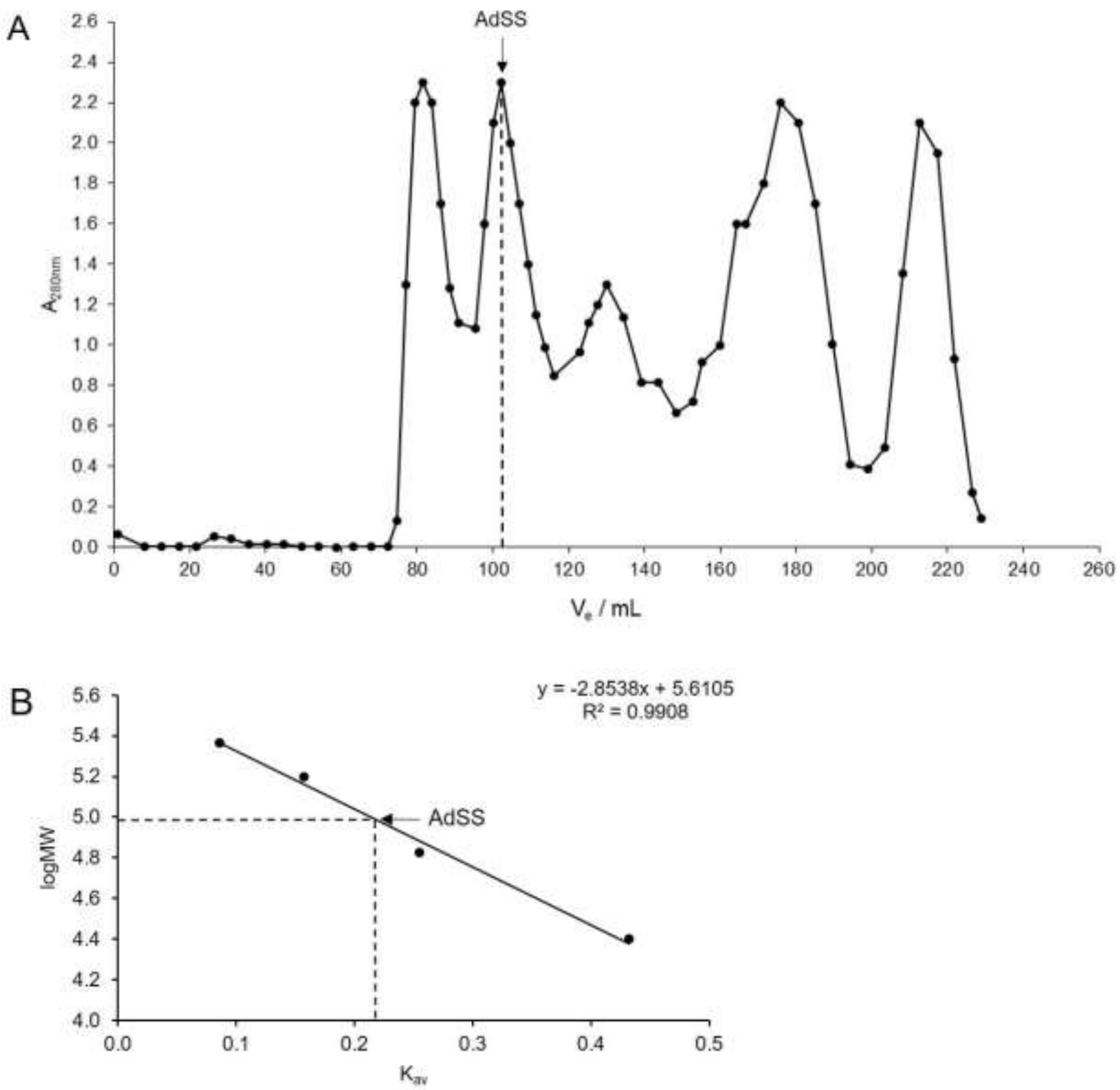

Figure 1Suppl. Estimation of *H. pylori* AdSS molecular mass by size-exclusion chromatography on Sephacryl S-200 column. A) Chromatogram; elution volume, V_e, for AdSS - 102 mL. B) Calibration curve for the used column; average distribution constant - $K_{av}=(V_e-V_0)/(V_c-V_0)$, V_0 – void volume of the column, V_c – geometrical bed volume of the column.

Additional reference:

47. Gorrell A, Wang W, Underbakke E, et al. Determinants of L-aspartate and IMP recognition in *Escherichia coli* adenylosuccinate synthetase. J Biol Chem 2002;277:8817–21.
